# Supplementary material for: Overcoming platinum-resistant ovarian cancer targeting the activated JAK-STAT pathways via extracellular vesicles
Source: Commun Biol. 2025 Aug 29;8:1305. doi: 10.1038/s42003-025-08771-9 (PMC12397317; doi:10.1038/s42003-025-08771-9)

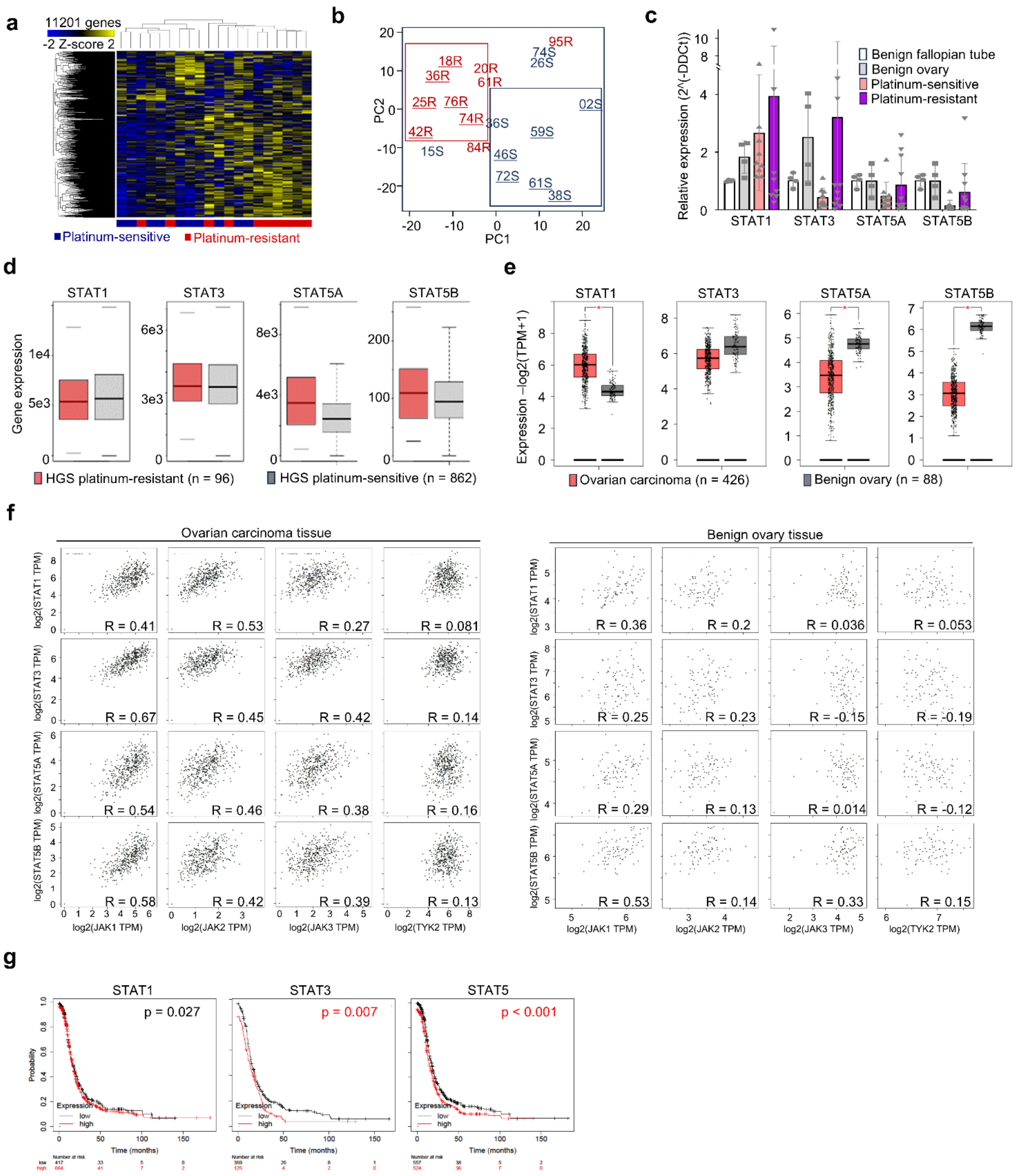

Supplementary Figure 1. Characterization of STAT-family expression in PROC.

a) Hierarchical clustering and heatmap demonstrating 11,201 differentially expressed genes between platinum-sensitive and resistant. The differentially expressed genes were defined as an absolute log2 fold change of >1.

b) Principal component analysis from bulk mRNA sequencing, including 10 platinum-resistant and 10 platinum-sensitive samples. Underlined 12 samples were selected for multivariate analysis.

c) STAT family expression in tissues by qRT-PCR, including 4 benign ovarian tubes, 4 benign ovaries, 10 platinum-sensitive HGSOC, and 10 resistant HGSOC tissue samples.

d) STAT family expression between platinum-resistant and platinum-sensitive HGSOC in 11 GEO and TCGA datasets using ROC Plotter.

e) STAT family expression between ovarian carcinoma and benign ovary using the GEPIA2 database.

f) JAK-STAT expression correlation in ovarian cancer tissue and benign ovarian tissue using the GEPIA2 database. R-values were calculated from Spearman's correlations.

g) Kaplan–Meier plots of disease-free survival according to STAT family expression in HGSOC using the Kaplan–Meier Plotter. *P*-values were calculated using the log-rank test.

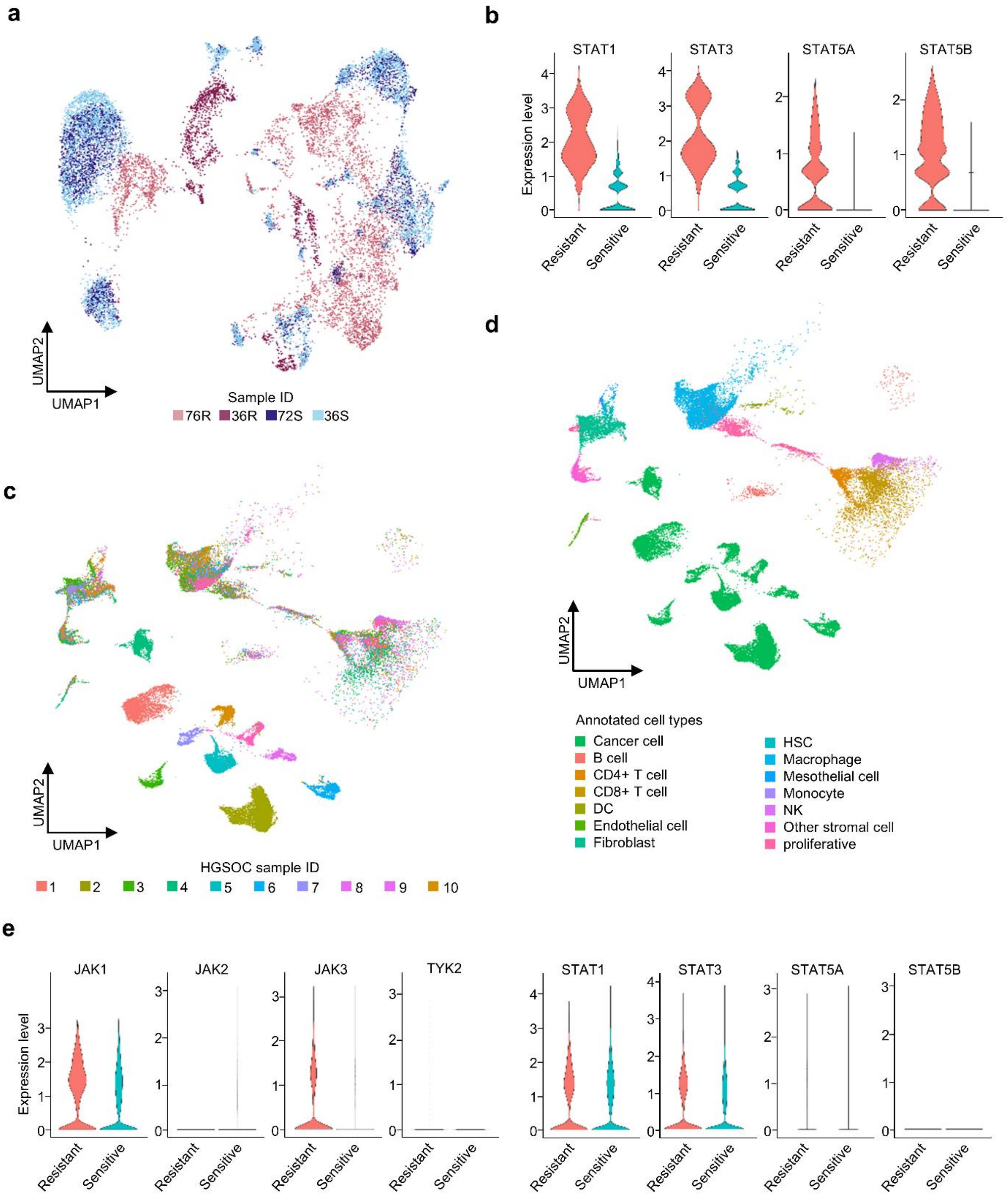

Supplementary Figure 2. JAK-STAT family gene expression in cancer cells.
a) UMAP plots of Visium spatial transcriptomics spots. Spatial transcriptomics spots were categorized into four clusters per sample.
b) Violin plot of STAT family expression in the cancer cell subpopulations.
c) UMAP plots demonstrating primary tumors (n = 10) from patients with HGSOC from the single-cell RNA sequencing dataset (PRJCA005422). Each dot was categorized into 10 clusters per sample, colored by cluster.
d) Each dot was categorized into 14 clusters of cell types, colored by cluster from the single-cell RNA sequencing dataset (PRJCA005422). DC: dendritic cell; HSC: hematopoietic stem cell; NK: natural killer.
e) Violin plot of JAK-STAT family expression in the cancer cell subpopulations from the single-cell RNA sequencing dataset (PRJCA005422).

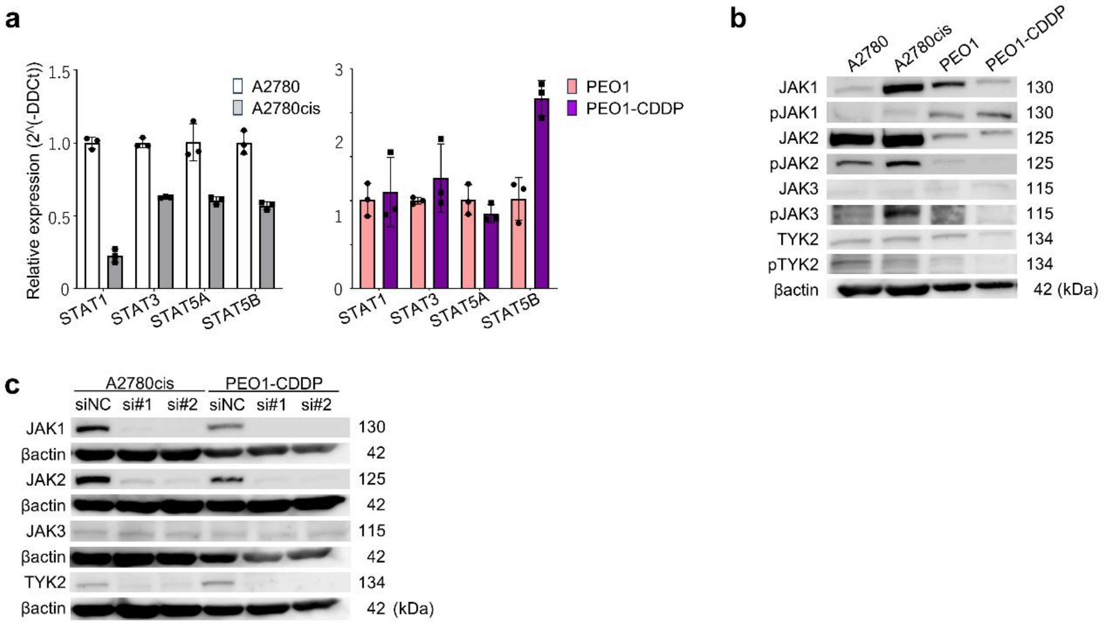

Supplementary Figure 3. JAK/STAT family expression in PROC cell lines.
a) Relative STAT family mRNA expression in paired parental and PROC cell lines. GAPDH was utilized as a reference gene to normalize expression. RNA extracted from individual biological replicates and plated in triplicate.
b) Immunoblotting analysis of whole-cell lysates from A2780, A2780cis, PEO1, and PEO1-CDDP cells, probed for JAK1, pJAK1, JAK2, pJAK2, JAK3, pJAK3, TYK2, pTYK2, and β-actin.
c) Evaluation of transfection efficiency of two siRNAs targeting the JAK family (siRNA No. 1 and No. 2). Western blot analysis of whole-cell lysates from A2780cis and PEO1-CDDP cells, probed for JAK1, JAK2, JAK3, TYK2, and β-actin.

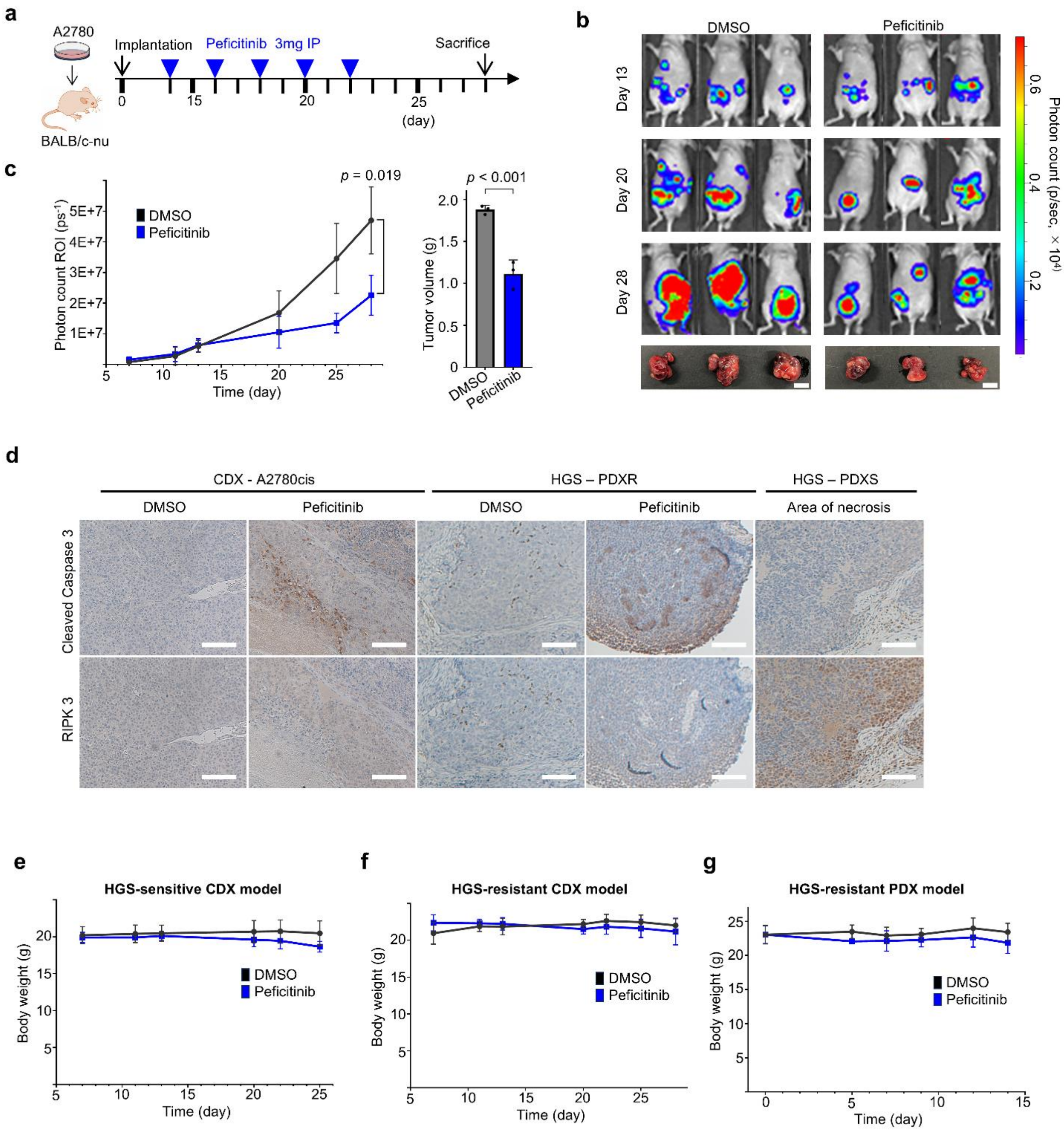

Supplementary Figure 4. Anti-tumor effects of JAK inhibitors in vivo.

a) Schema of the treatment schedule in A2780 platinum-sensitive cell line-derived xenograft model. Peficitinib or DMSO was administered intraperitoneally once every two days for a total of five injections.

b) Representative images of IVIS images and resected tumors. Photon values were measured once a week with an IVIS imaging system. Scale bars, 10 mm.

c) Quantitative analysis of tumor progression as bioluminescence images and resected tumors. Left: Relative photon counts of A2780-bearing mice treated with either DMSO or Peficitinib. Right: Mean tumor volume of A2780cis-bearing mice treated with either DMSO or Peficitinib. Relative photon count on day 28 and resected tumor volume were compared using Welch's t-test. (Peficitinib n = 3 mice; DMSO n = 3 mice)

d) Representative images of immunohistochemistry of cleaved caspase-3 and RIPK3 in the excised tumors (from Fig4. a, d and Supplementary Figure. 4a). Scale bars indicate 100 µm.

Body weight measurements in A2780-CDX **(e)**, A2780cis-CDX **(f)**, and PDX model **(g)** experiments.

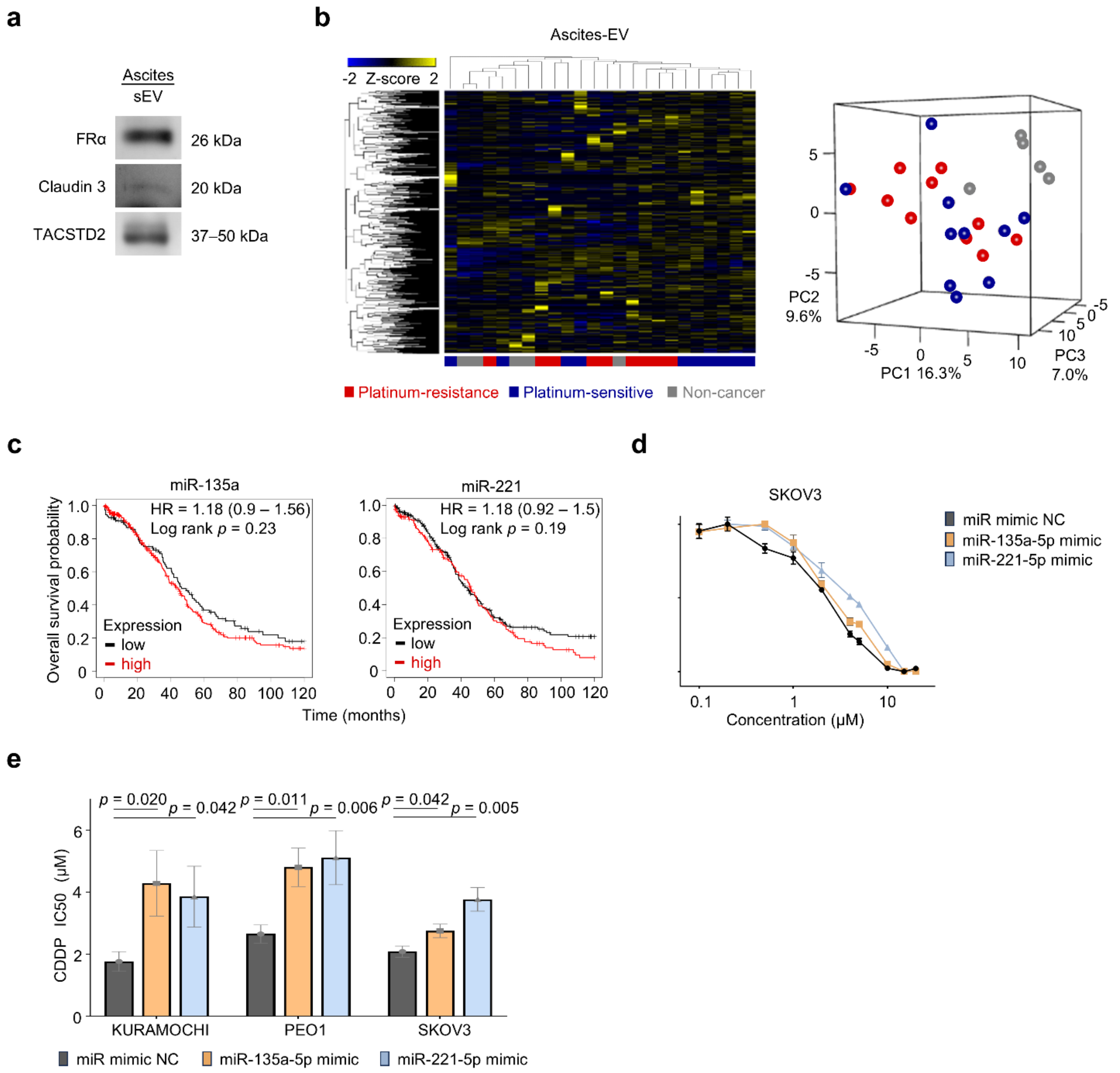

Supplementary Figure 5. Determining two miRNAs that are relevant in PROC.
a) Characterization of EVs from patient ascites. Immunoblot analysis for FRα, Claudin-3, and TACSTD2 of representative sEV samples.
b) Hierarchical clustering and heatmap expressed miRNA in ascites-EV of platinum-sensitive, platinum-resistant, and benign ascites. The differentially expressed miRNA were defined as an absolute log2 fold change exceeding principal component analysis of miRNA sequencing in ascites-EV, including 10 platinum-resistant, 10 platinum-sensitive, and 5 benign ascites samples.
c) Kaplan–Meier plots of overall survival according to miRNA expression in ovarian cancer tissue from the TCGA database using the Kaplan–Meier Plotter. *P*-values were calculated using the log-rank test.
d) Cisplatin sensitivity of transfected SKOV3 measured using the MTS assay. SKOV3 cells were transfected with a 20 nM-miRNA mimic for 24 h, and subsequently, cells were treated with cisplatin-containing medium for 48 h.
e) Cisplatin IC 50 values of KURAMOCHI, PEO1, and SKOV3 cells transfected with miRNA mimics, measured using the MTS assay.

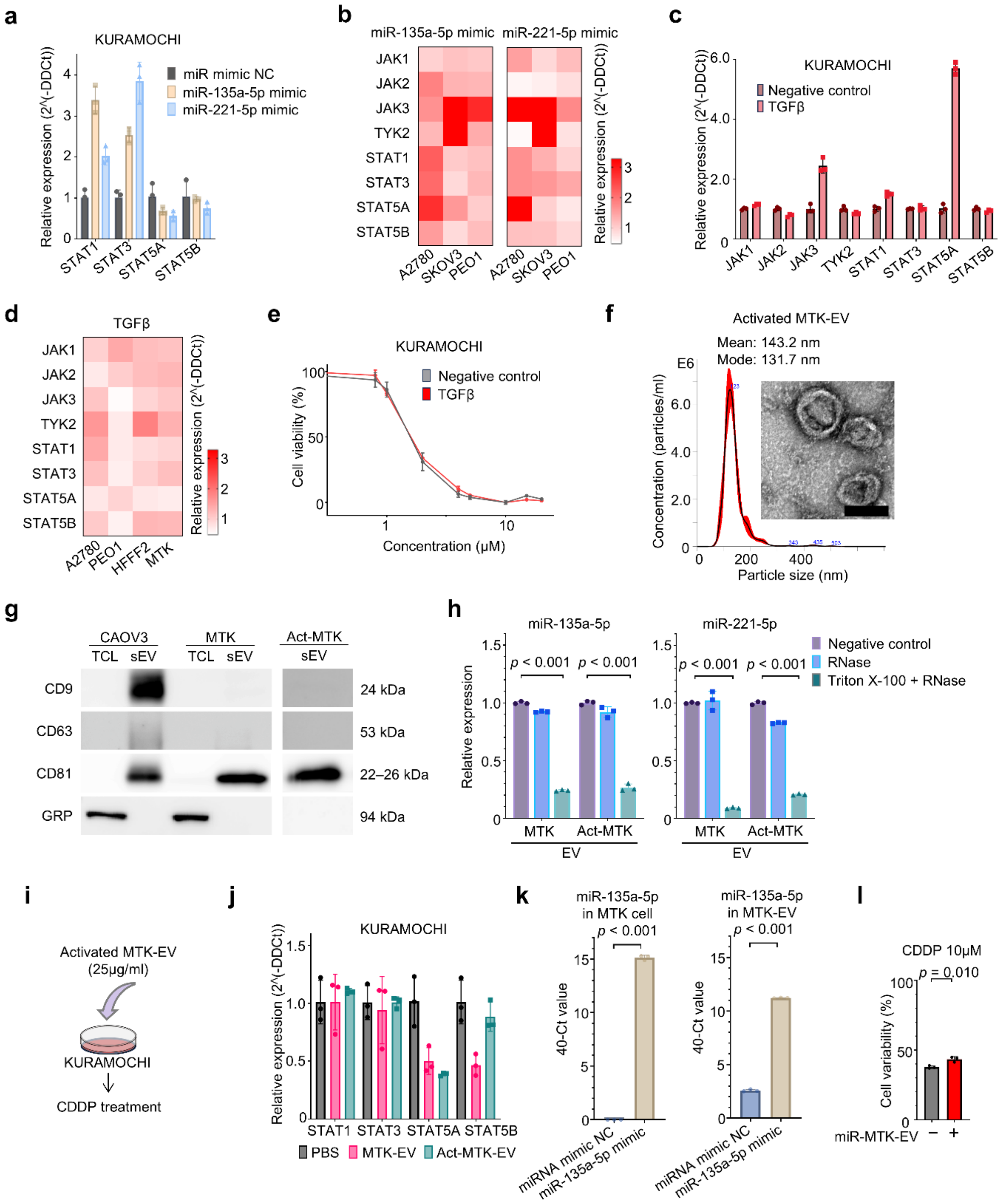

Supplementary Figure 6. Functional validation of two miRNAs with JAK inhibitors in PROC.

a) Relative STAT family mRNA expression in KURAMOCHI after transfection of miR-135a-5p and miR-221-5p. GAPDH was utilized as a reference gene to normalize expression. RNA extracted from individual biological replicates and plated in triplicate.

b) Relative JAK-STAT family mRNA expression of in A2780, SKOV3, and PEO1 after transfection of miR-135a-5p and miR-221-5p. Represents the relative expression levels to the miRNA-mimic negative control. GAPDH was utilized as a reference gene to normalize expression. RNA extracted from individual biological replicates and plated in triplicate.

c) Relative JAK-STAT family mRNA expression in KURAMOCHI after TGFβ exposure for 48 h. GAPDH was utilized as a reference gene to normalize expression. RNA extracted from individual biological replicates and plated in triplicate.

d) Relative JAK-STAT family mRNA expression in A2780, PEO1, HFFF2, and MTK after TGFβ exposure for 72 h. GAPDH was utilized as a reference gene to normalize expression. Represents the relative expression levels to the PBS as negative control.

e) Cisplatin sensitivity of KURAMOCHI cells measured using the MTS assay. KURAMOCHI cells were exposed to TGFβ for 48 h, and subsequently, cells were treated with cisplatin-containing medium for 72 h.

f) EV characterization from activated MTK cell culture medium. Nanoparticle tracking analyses demonstrating the particle size of the EVs. Transmission electron microscopy was utilized to visualize the EVs. The scale bar indicates 100 nm.
g) Immunoblot analysis for CD9, CD63, CD81, and GRP of cell lysate and sEV representative samples.
h) Relative expression of miR-135a-5p and miR-221-5p in EV treated with/ without RNaseA and/or Triton X-100.
i) Schematic protocol of KURAMOCHI cells treated with EVs. Activated MTK-EV derived from MTK cell cultures exposed to TGFβ for 48 h was administered to KURAMOCHI, followed by cisplatin treatment (CDDP).
j) Relative STAT family mRNA expression in KURAMOCHI following MTK-EV and activated MTK-EV treatment for 24 h. GAPDH was used as a reference gene to normalize expression. RNA extracted from individual biological replicates and plated in triplicate.
k) Expression of miR-135a-5p in MTK cell and MTK-EV after transfection of miR-135a-5p mimic for 24 h. RNA extracted from individual biological replicates and plated in triplicate. Student's t-test was utilized for comparison and p-values are presented in the graph.
l) Cell viability after cisplatin treatment of KURAMOCHI treated with/without miR-enriched MTK-EV (miR-MTK-EV) for 24 h, followed by cisplatin for 48 h

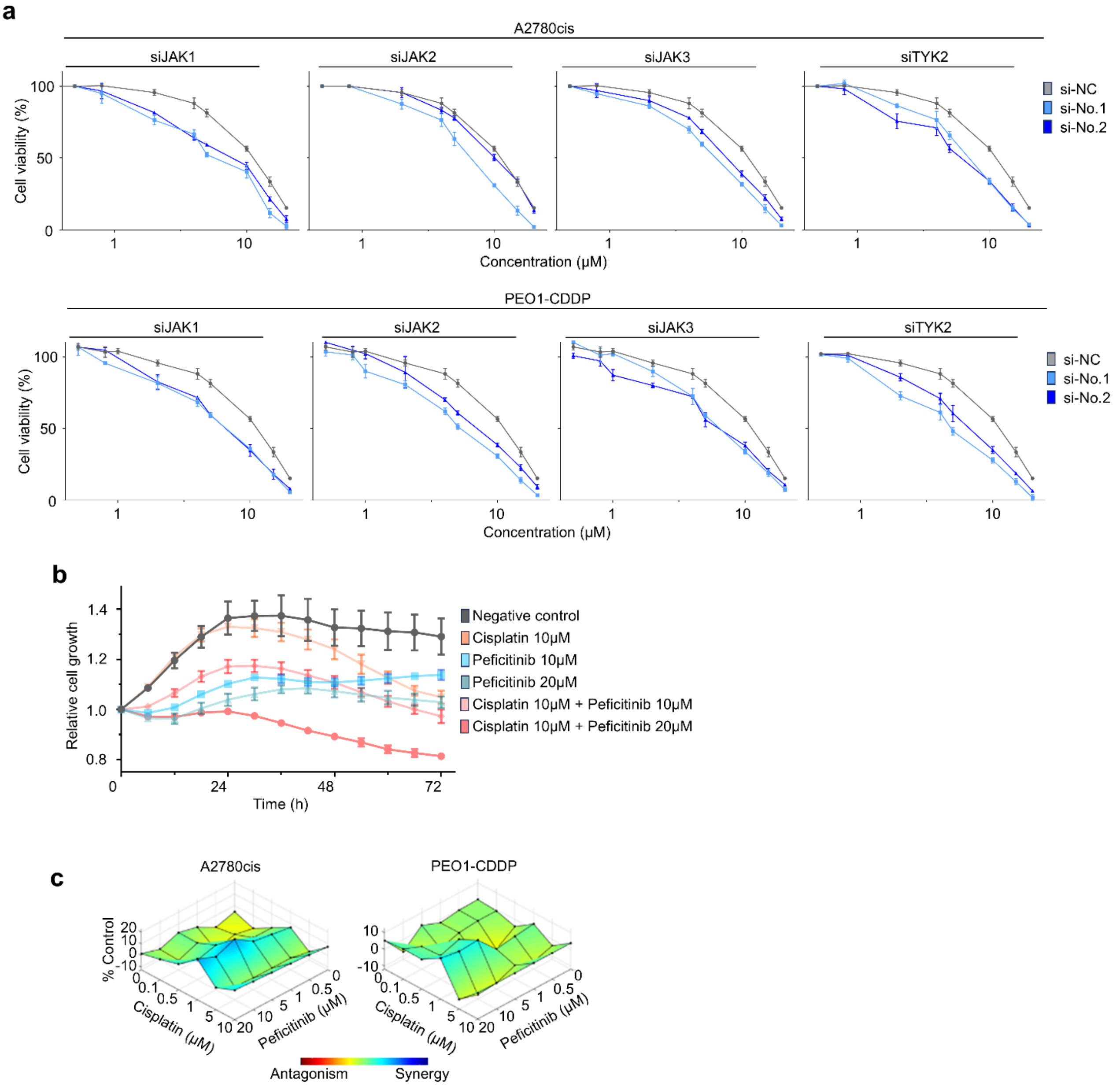

Supplementary Figure 7. Synergistic effect of peficitinib and cisplatin.

a) Cisplatin sensitivity of transfected A2780cis and PEO1-CDDP cells measured using the MTS assay. A2780cis and PEO1-CDDP cells were transfected with 3 nmol/L of each siRNA for 24 h, and subsequently, cells were treated with cisplatin-containing medium for 48 h.

b) Relative proliferation of A2780cis in peficitinib and/or cisplatin treatment. Cell viability was measured every 6 h.

c) Combination effect of peficitinib and cisplatin to A2780cis and PEO1-CDDP. A2780cis and PEO1-CDDP cells were treated with each drug concentration for 72 h, and the percentage of growth inhibition was demonstrated relative to untreated controls. Experiments were conducted in triplicate. Drug synergy was analyzed and graphical output was generated using Combenefit.

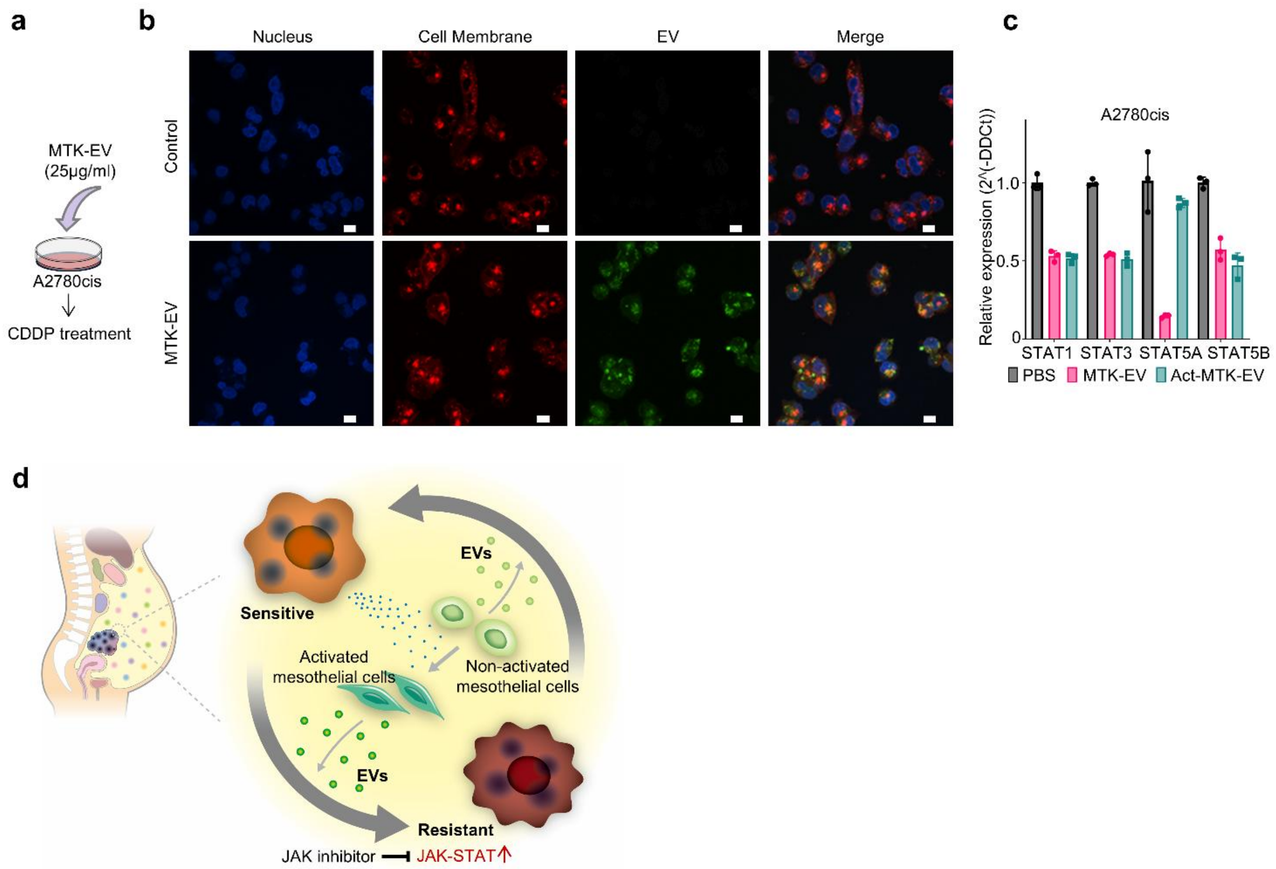

Supplementary Figure 8. Treatment of mesothelial cells derived EVs to PROC cell lines.

a) Schematic protocol of A2780cis cells treated with EVs. MTK-EV derived from MTK cell cultures was administered to A2780cis, followed by cisplatin treatment (CDDP).

b) Representative images of A2780cis cells taking up EVs from MTK cell culture medium using confocal laser scanning microscopy. The scale bar indicates 10 μm.

c) Relative STAT family mRNA expression in A2780cis after MTK-EV and activated MTK-EV treatment for 24 h. GAPDH was utilized as a reference gene to normalize expression. RNA extracted from individual biological replicates and plated in triplicate.

d) Schematic representation of the putative mechanism of platinum resistance by activated JAK-STAT pathways via miR-135a-5p enriched EVs derived from peritoneal mesothelial cells.

Supplementary Table 1.
Clinical cases of high-grade serous ovarian cancer used for miRNA-seq and bulk mRNA-seq analyses

Sample ID	Age	Clinical Stage	Primary treatment	Cytoreductive outcome	1stTFIp	PFS	OS	1st-line chemotherapy
HGS-18R	76	IIIC	NAC	Suboptimal	0	6	17	TC
HGS-36R	60	IIIC	NAC	Optimal	1	9	19	TC
HGS-42R	56	IIIC	PDS	Complete	1	6	16	TC
HGS-61R	64	IIIC	NAC	Optimal	2	7	23	TCB
HGS-25R	68	IVB	NAC	Optimal	3	11	32	TC
HGS-74R	71	IIIC	NAC	Complete	4	9	44	TC
HGS-84R	52	IIIC	NAC	Optimal	4	8	18	TC
HGS-76R	58	IIIC	NAC	Optimal	4	12	38	TC
HGS-95R	56	IVB	NAC	Optimal	5	13	32	TC
HGS-20R	48	IVB	NAC	Optimal	5	7	27	TC
HGS-15S	74	IIIC	PDS	Suboptimal	39	43	43	TC
HGS-02S	71	IVA	NAC	Suboptimal	40	49	49	TC
HGS-61S	75	IIIB	PDS	Optimal	44	48	48	TC
HGS-46S	72	IIIC	NAC	Complete	45	53	53	TC
HGS-26S	49	IIIB	PDS	Suboptimal	48	52	52	TC
HGS-72S	43	IIIC	PDS	Suboptimal	49	54	54	TC
HGS-36S	69	IVB	PDS	Complete	55	59	59	TC
HGS-74S	64	IIIC	NAC	Complete	60	67	67	TCB
HGS-59S	57	IIIB	PDS	Optimal	61	64	64	TC
HGS-38S	67	IIIC	NAC	Complete	65	74	74	TC

TFIp: Treatment-free interval of platinum
PFS: Progression-free survival
OS: Overall survival
NAC: Neoadjuvant chemotherapy
PDS: Primary debulking surgery
TC: Paclitaxel and carboplatin
TCB: Paclitaxel, carboplatin and bevacizumab

Supplementary Table 2.
List of oligos.

Name	Sequence (5'-3')	Note
JAK1 forward	ACAATACAGGGGAGCAGGTG	qRT-PCR
JAK1 reverse	TGCCGAGAACCCAAATAGTC	qRT-PCR
JAK2 forward	CCACTGGCCATCTATAACTC	qRT-PCR
JAK2 reverse	CTTTGCATTGGCTGAATTGC	qRT-PCR
JAK3 forward	AGTCCAACCTGATCGTGGTC	qRT-PCR
JAK3 reverse	ACCTTCAGCAGCACCTCTGT	qRT-PCR
TYK2 forward	CCTCCTGGAGATCTGCTTTG	qRT-PCR
TYK2 reverse	TCTGGGTTGGCTCATAGGTC	qRT-PCR
STAT1 forward	TTCAGGAAGACCCAATCCAG	qRT-PCR
STAT1 reverse	CCAGGCTCTTGATTTCATGC	qRT-PCR
STAT3 forward	TTTGTCAGCGATGGAGTACG	qRT-PCR
STAT3 reverse	GCTGCAACTCCTCCAGTTTC	qRT-PCR
STAT5A forward	GTTGGTCCTCTTGCCTCCTG	qRT-PCR
STAT5A reverse	GTCAAACCAGATCAGCTTTTACCC	qRT-PCR
STAT5B forward	CAAGGAGAACCTCGTGTTCC	qRT-PCR
STAT5B reverse	ACACTTCCATCACACCGTCA	qRT-PCR
GAPDH forward	CCAGGGCTGCTTTTAACTC	qRT-PCR
GAPDH reverse	GCTCCCCCCTGCAAATGA	qRT-PCR

Full unedited gel for Supplementary Figure 3b and 3c.

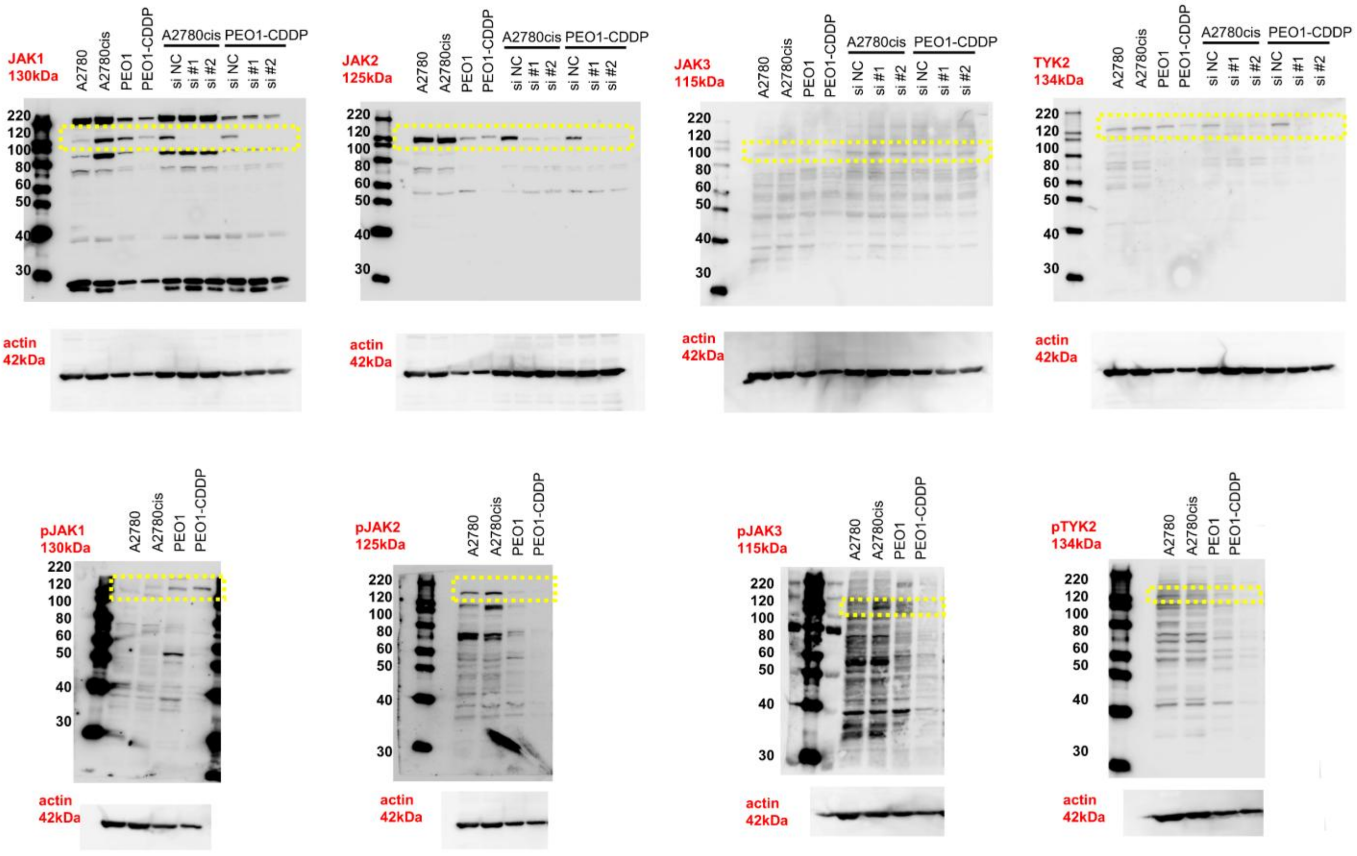

Full unedited gel for Supplementary Figure 5a.

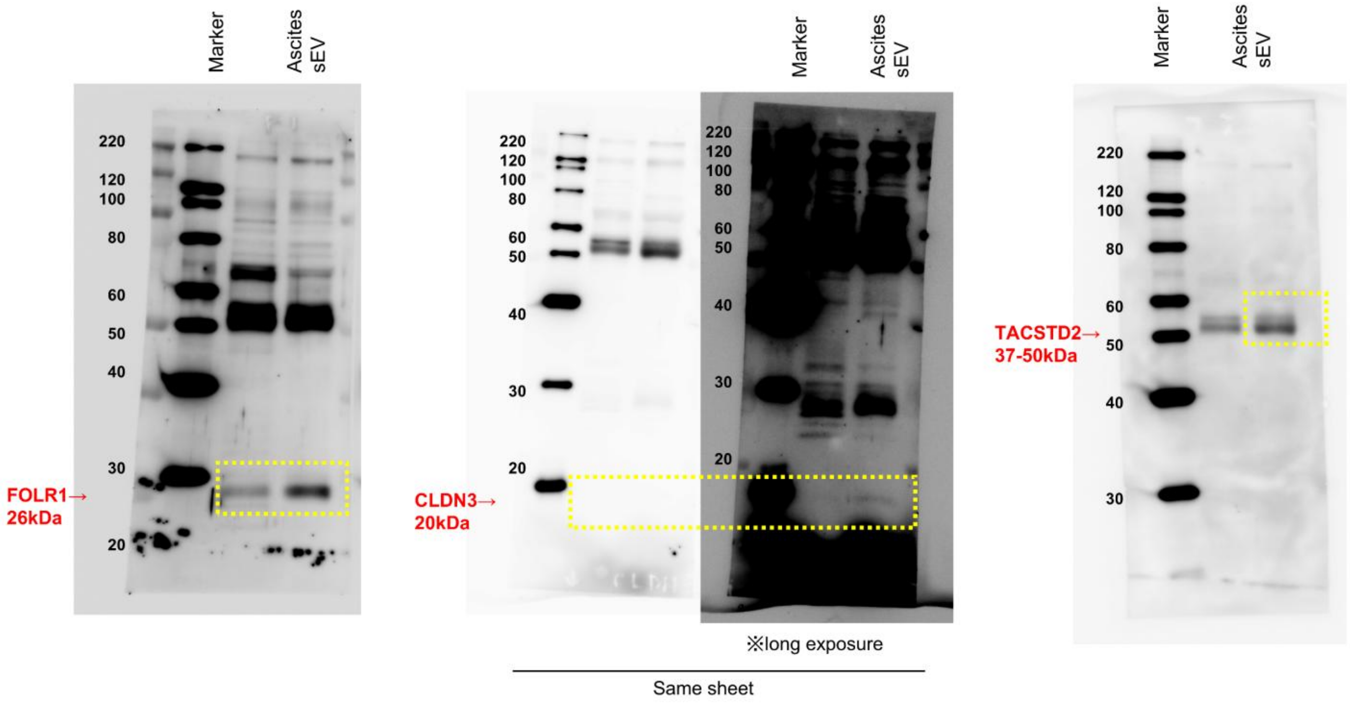

Full unedited gel for Figure 5b and Supplementary Figure 6g.

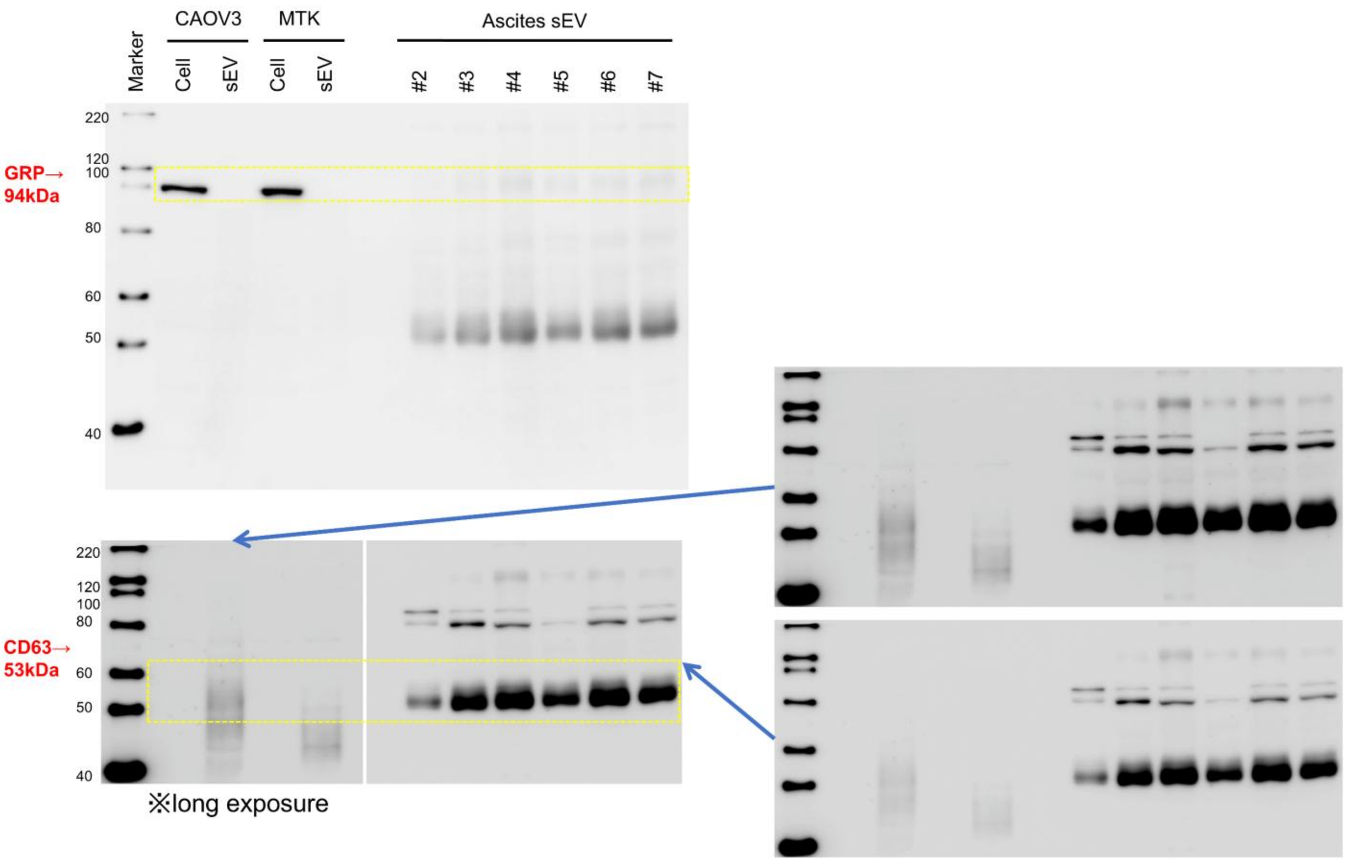

Full unedited gel for Figure 5b and Supplementary Figure 6g.

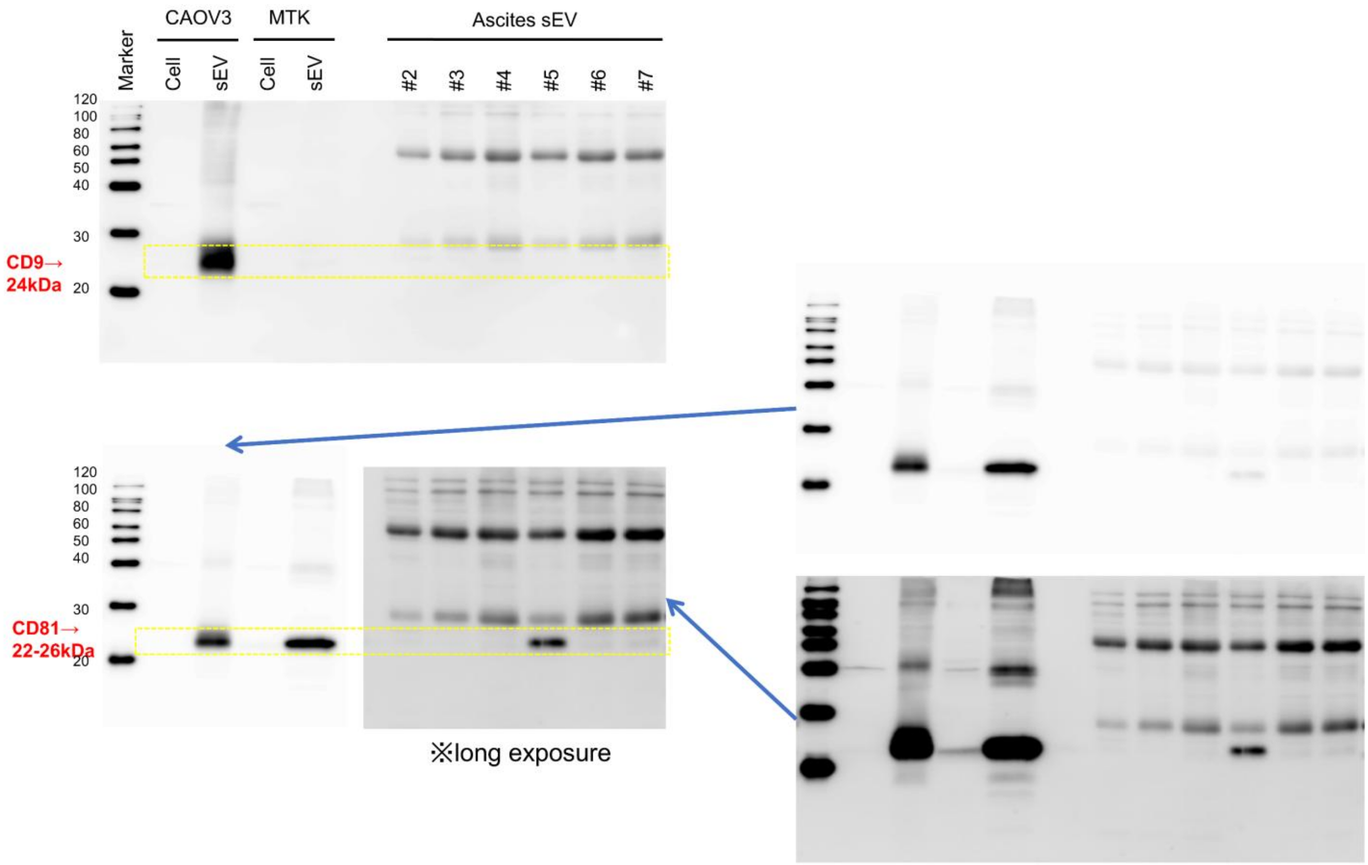

Supplement: Supplementary file 1 — Supplementary Materials [file 42003_2025_8771_MOESM1_ESM.pdf]
