# Supplementary material for: Overcoming platinum-resistant ovarian cancer targeting the activated JAK-STAT pathways via extracellular vesicles
Source: Commun Biol. 2025 Aug 29;8:1305. doi: 10.1038/s42003-025-08771-9 (PMC12397317; doi:10.1038/s42003-025-08771-9)
Supplement: Supplementary file 2 — Description of Additional Supplementary Files [file 42003_2025_8771_MOESM2_ESM.docx]

Description of Additional Supplementary Files

File name: Supplementary Data

Description: The source data behind all graphs in the paper.
